# The effect of Masai Barefoot technology (MBT) shoes on ankle joint complex kinematics and plantar heel pressure distribution

**DOI:** 10.1186/1757-1146-3-S1-P4

**Published:** 2010-12-20

**Authors:** Sophie Cox, Ivan Birch, Simon Otter

**Affiliations:** 1 University of Brighton, Brighton, UK

## Objectives

Footwear has long been a therapeutic option and recently MBT shoes have developed a shoe construction that claims to stimulate barefoot walking on soft, undulating ground. This study aimed to identify differences in range of motion at the ankle joint complex and plantar pressure distributions over the heel when walking in an MBT shoe compared with barefoot walking.

## Presentation content

To determine range of motion at the ankle joint complex the Cartesian Optoelectronic Dynamic Anthropometer (CODA mpx30) motion analysis system was used using 10 sensors attached to predefined anatomical landmarks using MBT sandals to prevent signal interference. Plantar pressure measurements were taken using the EMED SF pressure mat for barefoot pressures and the PEDAR in-shoe pressure analysis system and a 33% mask was applied to pressure data using Novel multimask software to capture the heel.

## Participant outcomes

Plantar pressure.

A total of 32 subjects participated in the study The mean maximum peak barefoot plantar pressure (432kPa ±SD 151) was significantly higher (p<0.001) than the mean maximum peak pressure in MBT shoes (225 kPa ±SD 38). The mean force-time integral was also significantly higher (p<0.001) during barefoot walking, 101 ±SD 27) than when walking in MBT shoes (70 ±SD 21).

Ankle joint complex kinematics

Both frontal plane and sagittal plane range of motion were significantly higher when wearing MBT shoes (p=0.013 and p=0.015 respectively). However, transverse plane motion was not significantly altered (p=0.47).

## Relevance

MBT shoes increase the range of motion at the ankle joint complex during gait and reduce both maximum peak pressure and force time integral at the heel in non pathological subjects

